# No Effects of Auditory and Visual White Noise on Oculomotor Control in Children with ADHD

**DOI:** 10.1177/10870547241273249

**Published:** 2024-09-09

**Authors:** Erica Jostrup, Emma Claesdotter-Knutsson, Pia Tallberg, Göran Söderlund, Peik Gustafsson, Marcus Nyström

**Affiliations:** 1 Lund University, Sweden; 2 Child and Adolescent Psychiatry Clinic, Region Skåne, Lund, Sweden; 3 Western Norway University of Applied Sciences, Sogndal, Norway; 4 University of Gothenburg, Sweden; 5 Lund University Humanities Lab, Sweden

**Keywords:** ADHD, white noise, eye tracking, inhibitory control, cognitive performance

## Abstract

**Background:**

White noise stimulation has demonstrated efficacy in enhancing working memory in children with ADHD. However, its impact on other executive functions commonly affected by ADHD, such as inhibitory control, remains largely unexplored. This research aims to explore the effects of two types of white noise stimulation on oculomotor inhibitory control in children with ADHD.

**Method:**

Memory guided saccade (MGS) and prolonged fixation (PF) performance was compared between children with ADHD (*N* = 52) and typically developing controls (TDC, *N* = 45), during auditory and visual white noise stimulation as well as in a no noise condition.

**Results:**

Neither the auditory nor the visual white noise had any beneficial effects on performance for either group.

**Conclusions:**

White noise stimulation does not appear to be beneficial for children with ADHD in tasks that target oculomotor inhibitory control. Potential explanations for this lack of noise benefit will be discussed.

## Introduction

The prevalence of ADHD among children is estimated to be approximately 5% globally, rendering it the most commonly diagnosed neurodevelopmental disorder in this population (Faraone et al., 2021). ADHD is characterized by developmentally inappropriate levels of inattention, hyperactivity, and/or impulsivity, significantly impacting daily functioning across educational, social, and family contexts (American Psychiatric Association, 2013). Interventions for ADHD encompass a wide range of strategies, including educational support, parent education, and pharmacological treatments such as stimulant or non-stimulant medication (Faltinsen et al., 2019).

While stimulant medication effectively mitigates behavioral symptoms associated with ADHD, its efficacy in addressing cognitive deficits related to ADHD remains uncertain (Tamminga et al., 2016). Executive function deficits, such as working memory deficits, increased reaction time variability, and response inhibition difficulties, are prominent cognitive markers of ADHD (Martinussen et al., 2005; Pievsky & McGrath, 2018). Despite receiving medication and educational support, children with ADHD often demonstrate academic underachievement, with a twofold increased risk of school dropout and eightfold higher likelihood of requiring special educational assistance (Fleming et al., 2017). Furthermore, medication adherence rates are low (Adler & Nierenberg, 2010; Brikell et al., 2024). Common side effects are insomnia, abdominal pain, and headache that further complicates treatment management (Sharma & Couture, 2014). In the World Federation of ADHD Consensus Statement, Faraone et al. (2021) highlights these issues by stating that while current treatments for ADHD exhibit efficacy, they are only moderately effective, emphasizing the need for research to “focus on biological and psychological causal mechanisms to find points of intervention that will improve the effectiveness of medical and non-medical treatments” (Faraone et al., 2021, p. 18).

Two recent reviews have highlighted the potential of auditory white noise stimulation as a non-pharmacological treatment that acts on cognitive performance in children with ADHD (Nigg et al., 2024; Pickens et al., 2019). White noise is a random signal that consists of frequencies with equal intensities, devoid of meaningful information (Oppenheim, 1999).

Studies on auditory white noise stimulation in children with ADHD have reported improvements in some areas of executive functioning, including verbal and spatial working memory, reading and writing speed, reduced no-go omissions, and reduced hyperactive behavior during stimulation (Baijot et al., 2016; Helps et al., 2014; Lin, 2022; Söderlund et al., 2016; Söderlund & Jobs, 2016). However, some tasks and cognitive abilities remain unaffected by noise exposure (Pickens et al., 2019). Similarly, typically developing children (TDC) have also demonstrated noise benefits across some, but not all, cognitive tasks (Awada et al., 2022; Lu et al., 2020; Othman et al., 2019; Rausch et al., 2014). In sum, prior research has primarily focused on the impact of auditory noise stimulation on working memory tasks, yielding somewhat conflicting results.

The working mechanism behind noise benefit is not known. There are few brain imaging studies on the effects of white noise stimulation, with somewhat different results. On the one hand, Baijot et al. (2016) found an increased Go P300 signal at Pz and improved performance in children with ADHD during white noise stimulation in a Go/No-go task. On the other hand, blink rate (a proxy for dopamine) was continuously higher in children with ADHD and not modulated by white noise. In addition, Rausch et al. (2014) have suggested that white noise exposure might increase activity in dopaminergic areas, such as the ventral tegmental area. Whether or not noise affects dopamine is thus not yet established but could be a potential explanation to why children with ADHD experience noise benefits.

The most prominent theory on why noise could be beneficial for cognitive performance is the moderate brain arousal (MBA) model (Sikström & Söderlund, 2007). The MBA model is based on stochastic resonance (SR), which is a phenomenon observed in threshold-based systems, like the nervous system. SR happens when weak signals are amplified by adding noise to the signal and thus making it stronger and, as a consequence, detectable (Moss et al., 2004). According to the MBA model moderate noise levels are believed to enhance cognitive performance the most. Too high or too low levels of noise are disruptive (Sikström & Söderlund, 2007). Further, the MBA model proposes that individuals with ADHD require higher levels of noise than typically developing individuals to achieve optimal performance (Sikström & Söderlund, 2007).

Although most research on noise stimulation thus far has been made on auditory noise, the MBA model and SR theory propose that noise stimulation should be efficient in any modality. Support for this notion has been found in a study on visual white pixel noise, which has shown improved reading performance in children with reading disability (Söderlund et al., 2021), a common comorbidity with ADHD. However, a recent study on the effects of stochastic vestibular stimulation on cognitive performance in children with ADHD found no improvements from the noise stimulation (Jostrup et al., 2023). The general ability of white noise stimulation to affect cognitive performance can thus be questioned and more research is needed to find under what conditions noise benefit occurs. Exploring the impact of noise stimulation on additional executive functions may offer valuable insights into the working mechanisms of white noise effects and consequently, the potential of noise stimulation as a non-pharmacological intervention for children with ADHD.

The objective of this study is to extend the research on white noise stimulation in two ways; (i) by exploring the effects of noise stimulation on other executive functions that are typically impaired in children with ADHD and, (ii) by comparing the effectiveness of two noise modalities.

Two recent meta-analyses have concluded that oculomotor abnormalities are robust symptoms of ADHD and thus may serve as promising biomarkers for ADHD (Chamorro et al., 2022; Maron et al., 2021). Children with ADHD are reported to have shorter saccadic latencies (Bucci et al., 2017), conduct more saccades during fixation tasks (Chamorro et al., 2022; Maron et al., 2021), perform more anticipatory saccades (Chamorro et al., 2022; Maron et al., 2021) and have difficulties initiating antisaccades, that is, saccades in the opposite direction of a stimuli (Bucci et al., 2017; Maron et al., 2021). These findings are suggested to correlate with deficiencies in inhibitory control (Bucci et al., 2017; Chamorro et al., 2022).

Impairments in inhibitory control are characterized by difficulties in suppressing nonproductive behaviors (i.e., impulsive actions, excessive fidgeting, interrupting others, and difficulty waiting for turns) and cognitive processing (i.e., impaired sustained attention, poor response inhibition, and challenges with task-switching), and have been associated with several neural dysfunctions in ADHD (Hart et al., 2013; Lukito et al., 2020; Norman et al., 2016). Although inhibitory control is a broad term, manual and oculomotor inhibitory control are suggested to be independent processes, with disruptions in oculomotor inhibitory control being a stronger predictor for impulsivity in ADHD (Roberts et al., 2011). Inhibitory control is closely associated with other executive functions, such as working memory, where white noise stimulation has demonstrated improvements. This provides reasons to assume that white noise stimulation could exert beneficial effects on oculomotor inhibitory control as well.

**Figure 1.**
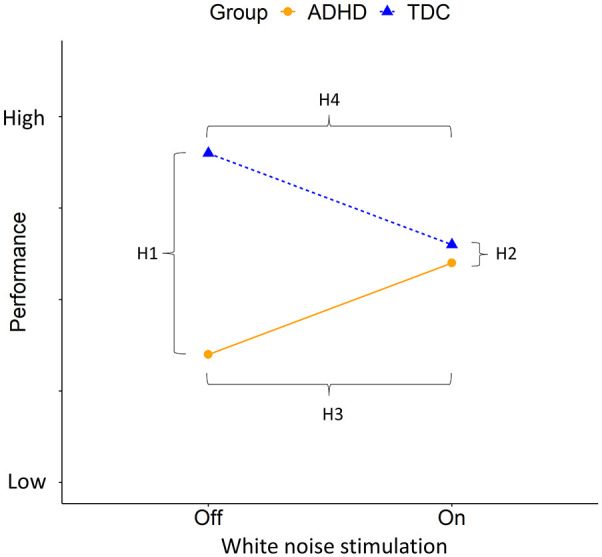
Hypotheses: H1: The TDC group will outperform the ADHD group in no noise while, H2: the difference between the groups will disappear during noise stimulation. H3: The ADHD group will experience increased performance from the noise stimulation while, H4: the TDC group will experience impaired performance from the noise stimulation.

This study investigates whether white noise stimulation in two modalities, auditory and visual, will have a positive effect on oculomotor control. A no noise condition will be used as a baseline. We compare the performance of oculomotor control during white noise stimulation between two groups, children with ADHD and TDC. Our hypotheses (Figure 1) stem from the MBA model and are as follows: (H1) there will be a difference in performance between the groups in the no noise condition. (H2) the difference between groups will disappear during noise stimulation. (H3) Children with ADHD will benefit from noise stimulation while (H4) TDC will be impaired by noise stimulation. According to the MBA model, the type of noise modality should not affect task performance differently.

## Method

### Design

A 2 × 2 × 4 design was used. Children diagnosed with ADHD were compared with typically developing children (TDC) on two tasks, a memory guided saccade task and a prolonged fixation task. These tasks were selected since they had the largest effect sizes in differentiating between children with ADHD and TDC in two meta-analyses from Chamorro et al. (2022) and Maron et al. (2021). The two tasks were performed in four different conditions: two different levels of visual white pixel noise, one level of auditory white noise and one no noise condition. PsychoPy (Peirce et al., 2019) code to run the experiment is publicly available on GitHub: https://github.com/marcus-nystrom/white-noise-exp.

### Participants

The study included 97 participants, aged between 7 and 16 years. These comprised 52 children with an ADHD diagnosis (31 boys and 21 girls, mean age = 11.7 years, *SD* = 1.7) and 45 TDC (20 boys and 25 girls, mean age = 11.7 years, *SD* = 2.4). The ADHD group was recruited from the outpatient child and adolescent psychiatry clinic in Lund, Sweden, while TDC were recruited from local schools in the district. With the goal to recruit a naturalistic sample for the study, that aimed to reflect the diversity found in both groups (Vos et al., 2022), as few exclusion criteria as possible were used. The inclusion criterion for TDC was an age-span between 7 and 16 years, while exclusion criteria included having a neurodevelopmental diagnosis.

Children with ADHD were diagnosed according to international guidelines following the Diagnostic and Statistical Manual of Mental Disorder, fifth Edition (DSM-5; American Psychiatric Association, 2013). Children with both combined, predominantly inattentive and predominantly hyperactive-impulsive type were included in the study. Senior consultants in child and adolescent psychiatry (two of the authors) confirmed all ADHD diagnoses. All children in the ADHD group, either awaiting medication initiation or on methylphenidate, were instructed to withdraw their medication 24 hours prior to participating in the study. Children 7 to 16 years old with ADHD diagnoses were eligible for the study. Medicating with other substance than methylphenidate and not complying with medication withdrawal were considered exclusion criteria.

Both groups were screened for hyperactivity and attention ability using the Swanson, Nolan and Pelham-IV (SNAP-IV) rating scale (Swanson et al., 2012). The SNAP-IV is a well-established parental and teacher rating scale assessing ADHD symptoms (18 items) and Oppositional Defiant Disorder (8 items) when screening for ADHD (Bussing et al., 2008) or evaluating treatment effect (Swanson et al., 2012). The symptom severity of each symptom item is rated on a four-point rating scale: 0 (not at all), 1 (just a little), 2 (quite a bit), and 3 (very much). The SNAP-IV enables the comparison of symptom severity across individuals and over time, aligning with the DSM-IV criteria for ADHD. The current study used the 18 items concerning ADHD symptoms. Parents rated each item based on the frequency or severity of observed behaviors. The screening aimed to validate the differences in symptoms between the ADHD and TDC groups.

The groups showed no significant differences in gender distribution or age. However, they exhibited statistically significant differences in hyperactivity, inattention, and total SNAP-IV score levels (Table 1).

**Table 1.** Participants’ Characteristics and Parent SNAP-IV Ratings on Hyperactivity and Inattention in the ADHD and TDC Groups.

Characteristics	ADHD	TDC	ADHD vs. TDC
Boy/girl	31/21	20/25	χ²(1, *N* = 97) = 1.66, *p* = .20
Age	11.7 (1.7)	11.7 (2.4)	*t*(79) = −0.00, *p* = .99
Hyperactivity	14.4 (6.3)	2.3 (3.3)	*t*(79) = −12.13, *p* < .001***
Inattention	18.4 (4.8)	3.7 (3.7)	*t*(94) = −17.02, *p* < .001***
Total (H+I)	32.8 (8.8)	6.0 (6.0)	*t*(91) = −17.74, *p* < .001***

*Note. M* (*SD*).

Age: years, H: hyperactivity, I: inattention.

*** *p* ≤ .001.

### Ethics

The study was granted ethical permission from the Swedish Ethical Review Authority (EPN 2023-02476-01) and was registered at Clinicaltrials.gov (NCT06057441). Written consent was obtained from all legal guardians of the participants, children aged 15 years or above also signed a written consent. Children under the age of 15 years approved verbally before participating in the study.

### Test Battery

#### Prolonged Fixation Task

**Figure 2.**
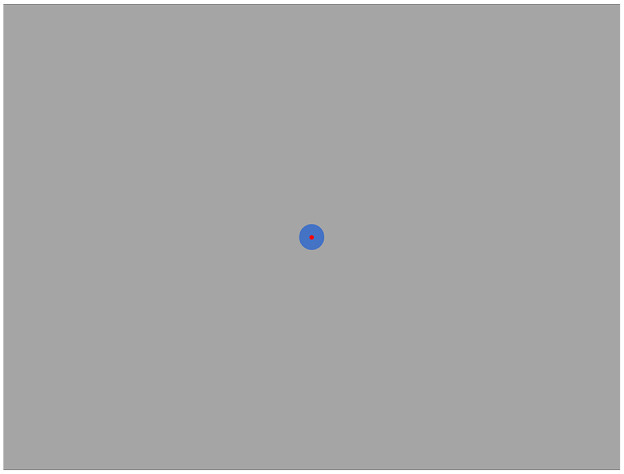
Stimulus for the prolonged fixation task, where participants were instructed to fixate the center of the blue/red dot for 60 seconds.

In the prolonged fixation (PF) task, participants were asked to fixate a point displayed in the center of the screen for 60 seconds. The fixation point consisted of a blue disc (1°) with a red disc (0.2°) in its center (Figure 2).

#### Memory Guided Saccade Task

In the memory guided saccade (MGS) task, the participant was asked to fixate on a central fixation point for as long as it was visible. During the time that the fixation point was visible, a white disc appeared in one of four different directions. The participant was instructed to not look at it but remember its location. After the central fixation point disappeared, the participants should move their gaze to the remembered location of the white disc as fast as possible. The disc would then reappear, and the participant could perform a corrective saccade to the correct location.

**Figure 3.**
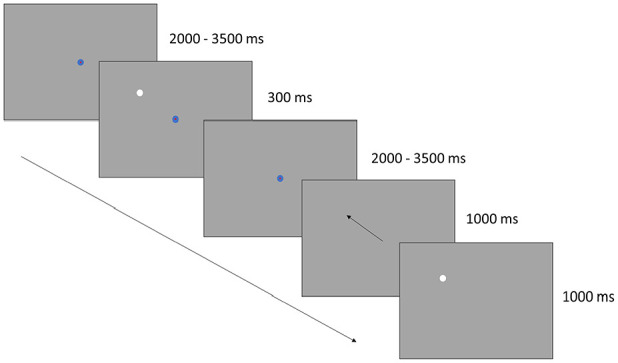
In the MGS task, participants fixated on a central point until it disappeared. Meanwhile, a white disc appeared in one of four directions, to which participants were instructed not to shift their gaze but to remember its location. Upon disappearance of the fixation point, participants were prompted to quickly redirect their gaze to the remembered location of the disc. Subsequently, the disc reappeared, allowing participants to correct any possible deviation between the gaze location and the disc location.

The central fixation point had the same appearance as in the PF task, that is a blue disc (1°) with a red disc (0.2°) in its center. In accordance with previous studies on MGS (Caldani et al., 2023; Mahone et al., 2009), the center point was visible for a random time interval (2,000–3,500 ms before disc onset, 2,000–3,500 ms after disc offset). The white disc (1°) randomly appeared in one of four directions (45°, 135°, 225°, or 315°) 10° away from the central fixation point and was visible for 300 ms. The white disc reappeared 1,000 ms after central fixation point offset and was visible for 1,000 ms (see Figure 3). Each participant completed 30 MGS-trials for each noise condition.

### Task Assessment

At the end of the experiment, participants were asked to rate how they experienced the tasks using a 10 cm visual analogue scale ranging from “fun” to “boring”.

### Intervention

#### Auditory White Noise

Auditory white noise was presented at 78 dB. It was generated from a uniform distribution U[0,225] as a stereo signal, that is, two arrays, one for each channel, were drawn from the distribution. The audio was sampled at 48,000 Hz, digitized to 16 bits, and saved as an audio file in uncompressed .wav-format. The noise level was calibrated separately for each computer with a UNI-T UT351/352 sound level meter before each experimental session.

#### Visual White Pixel Noise

**Figure 4.**
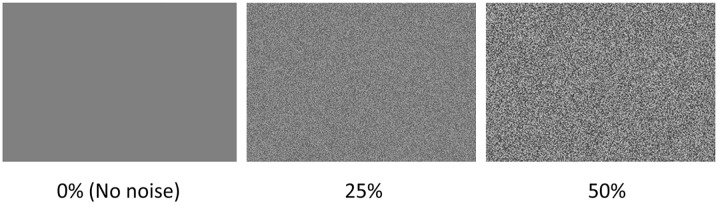
The visual white pixel noise at 0% (no noise), 25%, and 50%.

Two levels of visual white pixel noise were generated for the study, where the transparency of the noise image controlled the level of noise. The visual noise was added to each pixel in the stimulus image by blending it with a more or less transparent image of the same size with noise drawn from a uniform distribution U[0, 225]. In the distribution 0 represents black and 225 represents white. A noise level of 100% would result in only noise and no stimulus image whereas a noise level of 0% does not add any noise to the stimuli image at all. In this study we used the transparency levels of 25% and 50% (Figure 4). The noise was updated every screen refresh, at 60 Hz.

### Procedure

Before the start of the experiment, participants were introduced to the different types of noise and were instructed on how to perform the MGS task. Instructions like those of Mahone et al. (2009) were used: “As long as the center fixation point is visible, look only at the center point. Do not look at the flash when it occurs. When the center point disappears, then immediately move your gaze to the place where you saw the flash”. Participants also completed a training session of the MGS task, ensuring their understanding of it. The training assessed correct/incorrect trials and participants were only allowed to proceed to the experimental session after completing at least three correct trials, out of five. Correct trials were defined as maintaining the gaze within a 6° area from the central point until it disappeared and then moving the gaze to an area within 6° of where the white disc appeared. The training was performed without noise stimulation.

After successfully completing the training session, all participants performed two MGS tasks followed by four PF tasks and finally two MGS tasks. Each of the four noise conditions was randomly assigned to the tasks, such that all participants completed each task in all noise conditions. Between the first two MGS tasks and the PF tasks, participants had a planned break. During the break, instructions on how to perform the PF task were given. There was also a second planned break, between the PF tasks and the final MGS tasks. During the second break they were offered refreshments. The training and experimental session lasted about 50 minutes.

### Data Collection

Binocular eye movements were recorded with Tobii Pro Spectrum video-based eye trackers at 600 Hz (firmware: 2.6.1–orbicularis: 0) that outputs gaze coordinates in the screen coordinate system, where the upper left corner corresponds to (0,0) and the lower right corner corresponds to (1,1). Stimuli were presented with PsychoPy standalone version 2022.1.3 (Peirce et al., 2019), running Python version 3.8.10. Titta version 2.0.2 (Niehorster et al., 2020) was used for calibration and validation and TittaPy (version 1.0.0) was used for communication between PsychoPy and the eye tracker.

Data were collected in the Digital Classroom at Lund University Humanities Lab, a windowless room with lighting coming from the ceiling. The participants were placed 63 cm in front of a Eizo Flexscan EV2451 monitor with their head supported by a chin and forehead rest from Tobii Pro. During the entire experiment participants wore a pair of RØDE NTH-100M earphones, connected to a desktop computer running Windows 10.

The default calibration in Titta was used, with five calibration points and four points for validation. If accuracy were lower than 1° participants were recalibrated. If an accuracy higher than 1° was not reached within three calibrations, the best calibration was selected, and participants proceeded to training.

### Data Analysis

Fixations were detected with the Python implementation of the I2MC algorithm (Hessels et al., 2017), version 2.2.3, with default settings where both eyes were used as input. Accuracy and precision were computed from validation data (Niehorster et al., 2020). Data loss was computed from the data collected during the actual tasks. Saccades were defined as distances between two consecutive fixations.

To explore the hypothesized effect of white noise stimulation, separate analyses were conducted for each task. Two measures were computed from data collected during the PF task: fixation ratio and the number of intrusive saccades. Based on previous studies on memory-guided saccades (MGS; Bucci et al., 2018; Caldani et al., 2023), the MGS task was analyzed on three different measures, (i) the number of anticipatory saccades, (ii) the latency for correct trials and, (iii) the gain of correct trials. For this study we also added (iv) the ratio of correct trials.

#### Prolonged Fixation

##### Fixation Ratio

All fixations in the PF task were classified as correct or incorrect. To be classified as correct, the fixation had to be within a range of <2° from the central fixation point. The fixation ratio for each trial was then calculated as the duration of correct fixations divided by the duration of all fixations.

##### Intrusive Saccades

In the PF task, saccades with amplitudes larger than ≥2°were taken as intrusive saccades (Bucci et al., 2014, 2017, 2018). Saccades made in the beginning of the PF task to move the gaze to the central fixation point, were not included.

#### Memory Guided Saccades

##### Anticipatory Saccades

Anticipatory saccades are defined as saccades initiated before the extinction of the central fixation point and include saccades made <80 ms after the extinction of the central fixation point, since it takes at least 80 ms to react to the fact that the point has disappeared. The number of anticipatory saccades were counted as the number of saccades with amplitudes ≥2°. When analyzing anticipatory saccades, Caldani et al. (2023) reported the ratio of anticipatory saccades to correct saccades. In our analysis we analyze the number of anticipatory saccades instead of the ratio, since we argue that there is only one correct saccade.

##### Latency

Latency for each correct trial was calculated, that is, the time between the offset of the target and the onset of the next saccade ≥2°.

##### Gain

Gain, that is, the accuracy of saccades, was computed as the saccade amplitude divided by the target amplitude.

##### Correct Trials

Above analyses highlight specific aspects of the MGS task. To evaluate participants’ overall performance on the task, we also investigated the number of correct trials. To be classified as correct there had to (i) be at least one fixation between flash onset and target offset, (ii) the fixation(s) that were between flash onset and target offset had to be within a range of <2° from the central fixation point, (iii) there had to be at least an 80 ms latency after target offset, and no longer than 1,000 ms, until there was a new fixation. Additionally, (iv) the new fixation after target offset had to be ≥2° from the central fixation point and (v) the fixation had to be in the same quartile as the flash. The proportion between correct and incorrect trials were calculated. Fixations before flash onset were not analyzed.

### Statistical Analysis

The statistical analyses were done in R (R Core Team, 2022), version 4.1.3. A linear mixed effects model was fit, using lme4 (Bates et al., 2015), version 1.1-29, for each measure defined above. Independent variables for all models were noise condition, group and the interaction between noise condition and group. All models included a random intercept for each participant. Variance of the fitted models was then computed using the anova-function (Chambers & Hastie, 1992), which provides a type III analysis of variance table with Satterthwaite’s method. Significant main or interaction effects (*p* < .05) were further analyzed in post hoc tests: pairwise comparisons of estimated marginal means were done using the emmeans-function (Lenth, 2022), version 1.7.3, with Bonferroni correction.

All measures were checked for normality and homoscedasticity using visual inspection of residual plots. In PF task, the fixation ratio and intrusive saccades variables were transformed to normality using rank-based transformation. In the MGS task anticipatory saccades, latency and gain were skewed. Anticipatory saccades were transformed to normality with log-transformation after adding 1 to each trial, since the log(0) is undefined. Latency was transformed to normality using log-transformation and gain was transformed using square-root transformation.

Trials with >20% data loss from both eyes were excluded from the analysis. In total 14 trials (4 %) from 8 participants were excluded from the PF analysis. In the MGS task, trials with >20% data loss from both eyes between flash onset and reappearance of the flash was excluded. A total of 347 trials (3%) from 46 participants were excluded from the MGS analysis.

## Results

Data were first analyzed regarding precision, accuracy, and data loss, following the minimal reporting guideline for research involving eye tracking from Dunn et al. (2024). According to this guideline, precision is an estimate of uncertainty regarding the gaze position while accuracy is the offset between the estimation and true gaze position. Data loss refers to the proportion of invalid eye tracking data, due to for example, blinks or the participant looking outside the tracking range of the eye tracker (Dunn et al., 2024). Averaged across participants, precision in terms of sample-to-sample root mean square (RMS) distance was 0.07° (*SD* = 0.07°) for both right and left eye. Accuracy for left eye was 0.62° (*SD* = 0.24°) and 0.60° (*SD* = 0.27°) for right eye. Mean data loss was 4.67% (*SD* = 9.17%) for participants right eye and 4.92% (*SD* = 10.15%) for left eye. There were no significant differences between the groups in terms of accuracy and precision. However, a significant difference in data loss was observed, see Table 2.

**Table 2.** Precision, Accuracy, and Mean Data Loss in the ADHD and TDC Group.

Measure/eye	ADHD (*SD*)	TDC (*SD*)	ADHD vs. TDC *t*-statistics, *p*
Precision			
Right eye	0.08° (0.08°)	0.06° (0.04°)	*t*(73) = −1.48, *p* = .14
Left eye	0.08° (0.09°)	0.07° (0.03°)	*t*(60) = −1.03, *p* = .31
Accuracy			
Right eye	0.62° (0.29°)	0.58° (0.24°)	*t*(95) = −0.84, *p* = .41
Left eye	0.64° (0.27°)	0.60° (0.21°)	*t*(94) = −0.96, *p* = .34
Mean data loss			
Right eye	5.36% (10.19%)	3.87% (7.76%)	*t*(12,482) = −9.4, *p* < .001***
Left eye	5.22% (9.82%)	4.57% (10.50%)	*t*(12,171) = −3.6, *p* < .001***

*Note. M* (*SD*).

*** *p* < .001.

### Prolonged Fixation Task

#### Fixation Ratio

##### Auditory White Noise

**Figure 5.**
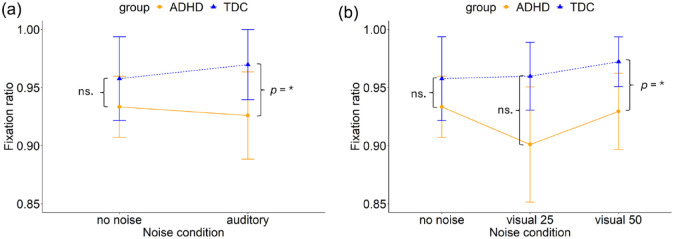
(a) Fixation ratio on the prolonged fixation task in the no noise and auditory white noise condition and (b) no noise and two levels of visual white pixel noise (25% and 50%) conditions for the ADHD and TDC group. Error bars represent 95% confidence intervals. *p < .05.

The linear mixed effects model, fitted with a two-way repeated measures ANOVA (2 groups × 2 noise conditions), revealed no main effect of noise (*F*(1, 95) = 3.01, *p* = .086) on fixation ratio. However, we observed a significant main effect of group (*F*(1, 95) = 4.26, *p* = .042). There was no interaction between noise condition and group (*F*(1, 95) = 0.20, *p* = .655), see Figure 5a. The model explained 4.0% of the variance (marginal *R*²) and 55.7% of the variance when accounting for both fixed and random effects (conditional *R*²).

A post hoc analysis of group differences found no difference between the ADHD group (ADHD: *M* = 0.93, *SD* = 0.09) and TDC group (TDC: *M* = 0.96, *SD* = 0.12) in the no noise condition (β = − 17.9 (*SE* = 11.2), *t*(147) = −1.6, *p* = .113). However, a significant difference between the groups in the auditory noise condition (β = − 22.8 (*SE* = 11.2), *t*(147) = −2.0, *p* = .045) was found. The ADHD group had a significantly lower fixation ratio (*M* = 0.93, *SD* = 0.13) compared to the TDC group (*M* = 0.97, *SD* = 0.10).

##### Visual White Pixel Noise

A two way repeated measures ANOVA (2 groups × 3 noise conditions) found no main effect of noise (*F*(2, 190) = 1.90, *p* = .153) on fixation ratio. However, a significant main effect of group (*F*(1, 95) = 4.70, *p* = .033) was observed. There was no interaction between noise condition and group (*F*(2, 190) = 0.13, *p* = .880), see Figure 5b. The model explained 3.9% of the variance (marginal *R*²) and 58.2% of the variance when accounting for both fixed and random effects (conditional *R*²).

A post hoc analysis found no differences between the ADHD and the TDC group in the no noise condition (β = −26.8 (*SE* = 16.8), *t*(174) = −1.6, *p* = .113), the groups did not differ in their fixation ratio (ADHD: *M* = 0.93, *SD* = 0.09; TDC: *M* = 0.96, *SD* = 0.12). In the visual noise condition at level 25% no difference between the groups was found (β = −30.6 (*SE* = 16.8), *t*(174) = −1.8, *p* = .071)he ADHD group did not differ in their fixation ratio (*M* = 0.90, *SD* = 0.17) compared to the TDC group (*M* = 0.96, *SD* = 0.10). In the noise condition at level 50%, a significant difference between the groups was revealed (β = −34.8 (*SE* = 16.8), *t*(174) = −2.1, *p* = .040). The ADHD group had a significantly smaller fixation ratio (*M* = 0.93, *SD* = 0.12) compared to the TDC group (*M* = 0.97, *SD* = 0.07).

#### Intrusive Saccades

##### Auditory White Noise

**Figure 6.**
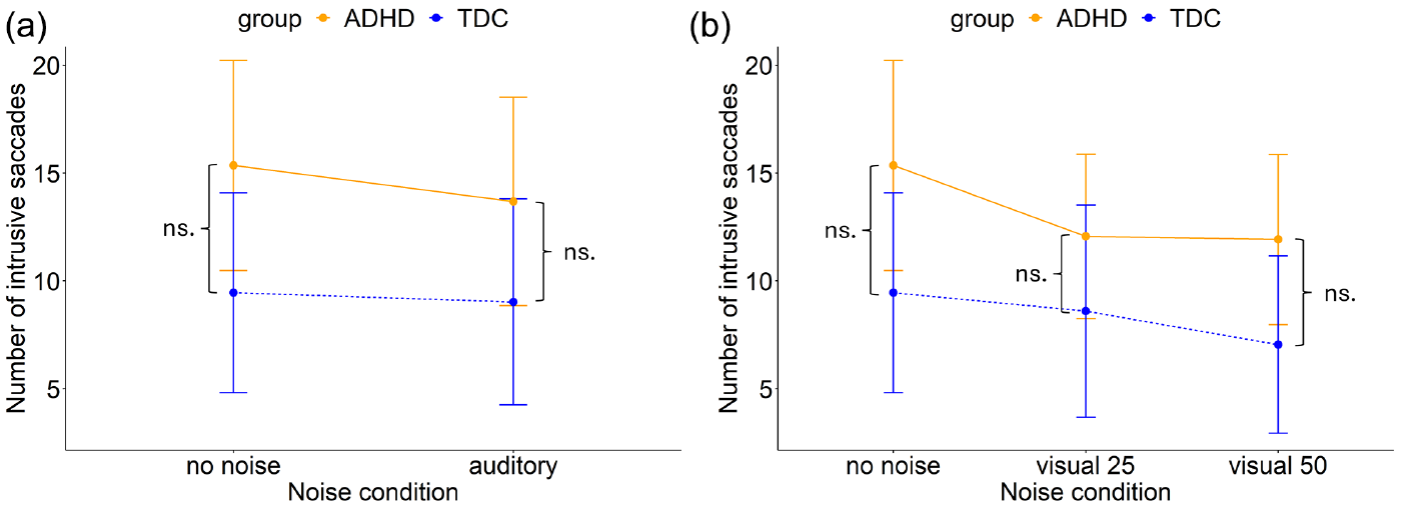
Number of intrusive saccades for the ADHD and TDC group in the prolonged fixation task in (a) no noise and auditory noise, and (b) no noise, visual noise at 25% and visual noise at 50%. Error bars represent 95% confidence intervals.

A two way repeated measures ANOVA revealed no main effect of noise (*F*(1, 95) = 1.52, *p* = .221) or group (*F*(1, 95) = 3.11, *p* = .081). No interaction between noise and group (*F*(1, 95) = 0.03, *p* = .856) was found, see Figure 6a. The groups did not differ in the number of intrusive saccades made during the prolonged fixation task in the no noise condition (ADHD: *M* = 15.35, *SD* = 17.33; TDC: *M* = 9.46, *SD* = 15.22) and the auditory noise condition (ADHD: *M* = 13.68, *SD* = 17.01; TDC: *M* = 9.02, *SD* = 15.71). The model explained 2.8% of the variance (marginal *R*²) and 64.3% of the variance when accounting for both fixed and random effects (conditional *R*²).

##### Visual White Pixel Noise

A two way repeated measures ANOVA revealed no main effect of noise (*F*(2, 190) = 2.67, *p* = .072). However, we observed a trend toward a significant main effect of group (*F*(1, 95) = 3.90, *p* = .051). No interaction between noise and group (*F*(2, 190) = 0.24, *p* = .788) was found, see Figure 6b. The groups did not statistically differ in the number of intrusive saccades in the no noise condition (ADHD: *M* = 15.35, *SD* = 17.33; TDC: *M* = 9.46, *SD* = 15.22), the visual noise at level 25% condition (ADHD: *M* = 12.06, *SD* = 13.11; TDC: *M* = 8.61, *SD* = 15.99), and the visual noise at level 50% condition (ADHD: *M* = 11.92, *SD* = 14.02; TDC: *M* = 7.05, *SD* = 13.35). The model explained 3.7% of the variance (marginal *R*²) and 64.5% of the variance when accounting for both fixed and random effects (conditional *R*²). Overall, the noise stimulation did not significantly impact the performance of either group in terms of the number of intrusive saccades.

### Memory Guided Saccade Task

#### Anticipatory Saccades

##### Auditory White Noise

**Figure 7.**
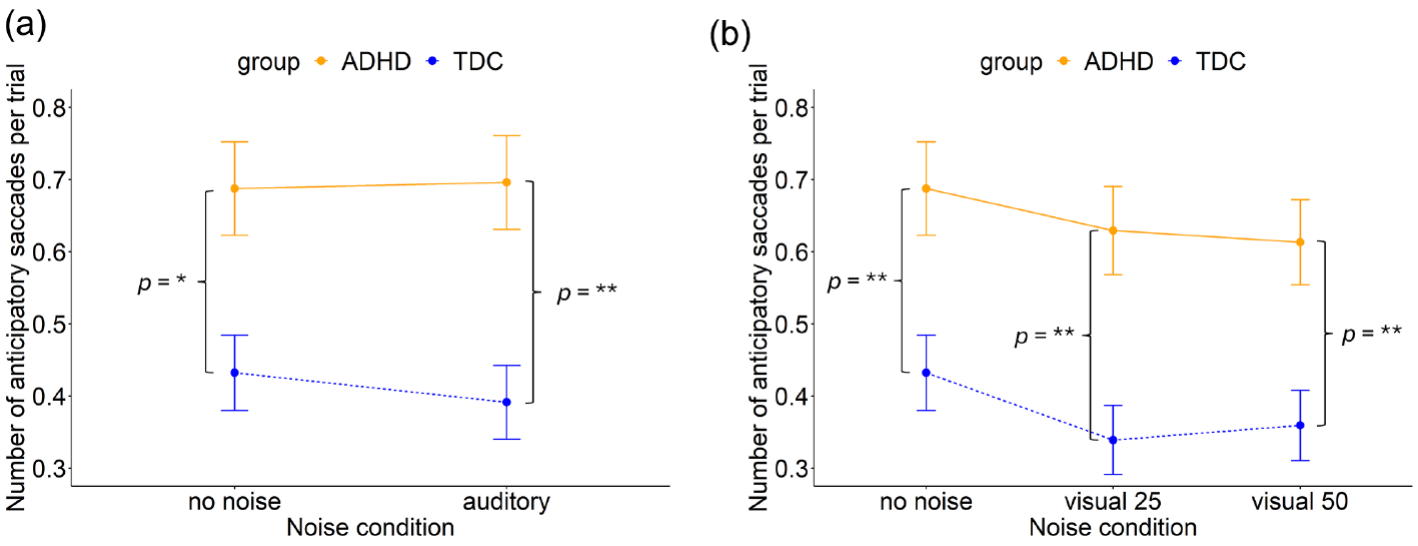
Number of anticipatory saccades per trial in the ADHD and TDC group during (a) no noise and auditory noise and, (b) no noise, visual white pixel noise at 25% and visual white pixel noise at 50%. Error bars represent 95% confidence intervals. *p < .05. **p < .01.

The ANOVA found no main effect of noise (*F*(1, 5,570) = 1.23, *p* = .268). However, we observed a significant main effect of group (*F*(1, 95) = 8.11, *p* = .005). No interaction between noise and group (*F*(1, 5,570) = 0.89, *p* = .346) was found, see Figure 7a. The model explained 1.6% of the variance (marginal *R*²) and 18.5% of the variance when accounting for both fixed and random effects (conditional *R*²).

A post hoc analysis indicated a significant difference between the groups in both the auditory noise condition (β = .14 (*SE* = 0.05), *z* = 3.0, *p* = .003) and the no noise condition (β = .12 (*SE* = 0.05), *z* = 2.5, *p* = .013). The ADHD group made significantly more anticipatory saccades in both the no noise condition (ADHD: *M* = 0.69, *SD* = 1.28; TDC: *M* = 0.43, *SD* = 0.97) and the auditory noise condition (ADHD: *M* = 0.70, *SD* = 1.28; TDC: *M* = 0.39, *SD* = 0.96) compared to the TDC group.

##### Visual White Pixel Noise

The ANOVA revealed a significant main effect of noise (*F*(2, 8,357) = 4.16, *p* = .016) and a significant main effect of group (*F*(1, 93) = 8.31, *p* = .005). No interaction between noise and group (*F*(2, 8,357) = 0.10, *p* = .907) was found, see Figure 7b. The model explained 1.4% of the variance (marginal *R*²) and 16.4% of the variance when accounting for both fixed and random effects (conditional *R*²).

A post hoc analysis indicated a significant difference between the groups across all noise conditions, both the no noise condition (β = .12 (*SE* = 0.04), *z* = 2.7, *p* = .007), the visual noise condition at level 25% (β = .12 (*SE* = 0.04), *z* = 2.9, *p* = .004) and visual noise condition at level 50% (β = .11 (*SE* = 0.04), *z* = 2.6, *p* = .009). The ADHD group made significantly more anticipatory saccades in both the no noise condition (ADHD: *M* = 0.69, *SD* = 1.28; TDC: *M* = 0.43, *SD* = 0.97), the visual noise condition at level 25% (ADHD: *M* = 0.63, *SD* = 1.21; TDC: *M* = 0.34, *SD* = 0.88), and the visual noise condition at level 50% (ADHD: *M* = 0.61, *SD* = 1.16; TDC: *M* = 0.36, *SD* = 0.90) compared to the TDC group.

However, post hoc tests did not detect any significant differences between the noise conditions within groups (all *p* > .05). This discrepancy, from the significant main effect found in the ANOVA, suggests that factors other than specific pairwise comparisons may drive the observed overall differences among the noise conditions.

#### Latency

##### Auditory Noise

**Figure 8.**
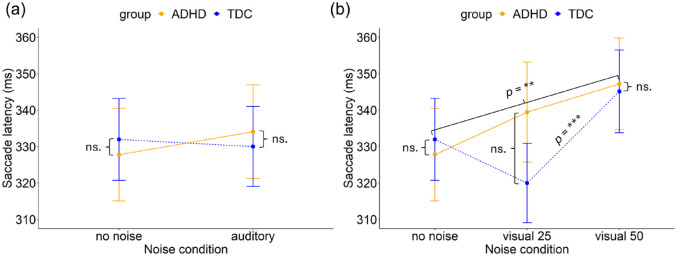
Saccade latency for correct trials for the ADHD and TDC group in (a) no noise and auditory noise and, (b) no noise, visual white pixel noise at 25% and visual white pixel noise at 50%. Error bars represent 95% confidence intervals. **p < .01. ***p < .001.

An ANOVA found no main effect of noise (*F*(1, 3,067) = 0.09, *p* = .771) or group (*F*(1, 95) = 0.33, *p* = .566) and no interaction between noise and group (*F*(1, 3067) = 0.86, *p* = 0.354) in saccadic latency, see Figure 8a. The model explained 1.0% of the variance (marginal *R*²) and 15.5% of the variance when accounting for both fixed and random effects (conditional *R*²).

##### Visual Noise

In the visual noise condition, an ANOVA found a significant main effect of noise (*F*(2, 4457) = 7.52, *p* < .001). No main effect of group (*F*(1, 93) = 0.09, *p* = .770) was found. However, a significant curve linear interaction between noise and group (*F*(2, 4,457) = 3.79, *p* = .023) was shown, see Figure 8b. The model explained 0.5% of the variance (marginal *R*²) and 15.5% of the variance when accounting for both fixed and random effects (conditional *R*²).

A post hoc analysis revealed specific effects of noise condition on latency for both the ADHD and TDC groups. The ADHD group exhibited a significant increase in latency in the visual noise condition at 50% (*M* = 347.2 ms, *SD* = 170.5 ms) compared to the no noise condition (*M* = 327.8 ms, *SD* = 174.5 ms; β = -.07 (*SE* = 0.02), *z* = −3.1, *p* = .006). There was no significant difference in latency between the visual noise condition at level 25% (*M* = 339.5 ms, *SD* = 178.0 ms) and the other noise conditions for the ADHD group.

Similarly, the TDC group demonstrated a significant increase in latency in the visual noise condition at 50% (*M* = 345.1 ms, *SD* = 164.4 ms) compared to the visual white noise condition at level 25% (*M* = 320.0 ms, *SD* = 159.1 ms; β = -.07 (*SE* = 0.02), *z* = −3.7, *p* < .001). There was no significant difference between the no noise condition (*M* = 332.0 ms, *SD* = 163 ms) and the other noise conditions for the TDC group.

#### Gain

##### Auditory White Noise

**Figure 9.**
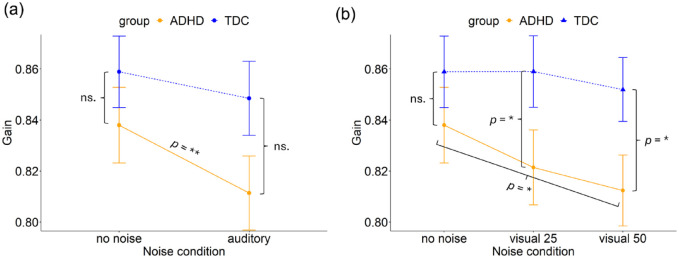
Gain of memory guided saccades for the ADHD and TDC groups in the (a) no noise and auditory noise condition and, (b) no noise, visual white pixel noise at 25% and visual white pixel noise at 50% conditions. Error bars represent 95% confidence intervals. *p < .05. **p < .01.

In the analysis of gain, the ANOVA found a significant main effect of noise (*F*(1, 3,060) = 9.82, *p* = .002). No main effect of group (*F*(1, 84) = 2.54, *p* = .115) and no interaction between noise and group (*F*(1, 3,060) = 1.48, *p* = .225) was found, see Figure 9a. The model explained 0.8% of the variance (marginal *R*²) and 16.2% of the variance when accounting for both fixed and random effects (conditional *R*²).

A post hoc analysis indicated a significant decline in gain between the no noise condition (*M* = 0.84, *SD* = 0.21) and the auditory noise condition (*M* = 0.81, *SD* = 0.20; β = .02 (*SE* = 0.01), *z* = 3.0, *p* = .003) in the ADHD group. No effect of noise was found in the TDC group, they did not differ in gain between the no noise condition (*M* = 0.86, *SD* = 0.20) and the auditory noise condition (*M* = 0.85, *SD* = 0.21).

##### Visual White Pixel Noise

The ANOVA revealed a significant main effect of noise (*F*(2, 4,459) = 3.79, *p* = .023) and a significant main effect of group (*F*(1, 93) = 4.24, *p* = .042). No interaction between noise and group (*F*(2, 4,459) = 1.45, *p* = .236) was found, see Figure 9b. The model explained 0.9% of the variance (marginal *R*²) and 14.8% of the variance when accounting for both fixed and random effects (conditional *R*²).

Post hoc analyses revealed significant effects of noise within the ADHD group, indicating a significant decrease in gain between the no noise condition and the visual noise at level 50% condition (β = .02 (*SE* = .01), *z* = 2.9, *p* = .011).

Furthermore, significant differences were observed between the ADHD and TDC groups during visual white noise at level 25% (ADHD: *M* = 0.82, *SD* = 0.19; TDC: *M* = 0.86, *SD* = 0.20; β = -.02 (*SE* = 0.01), *z* = −2.2, *p* = .029) and visual white noise at level 50% (ADHD: *M* = 0.81, *SD* = 0.19; TDC: *M* = 0.85, *SD* = 0.18; β = -.02 (*SE* = 0.01), *z* = −2.3, *p* = .025), but not in the no noise condition (ADHD: *M* = 0.84, *SD* = 0.21; TDC: *M* = 0.86, *SD* = 0.20; β = -.01 (*SE* = 0.01), *z* = −1.1, *p* = .255). The ADHD group had a significantly lower gain than the TDC group across noise conditions.

#### Correctly Performed Trials

##### Auditory Noise

**Figure 10.**
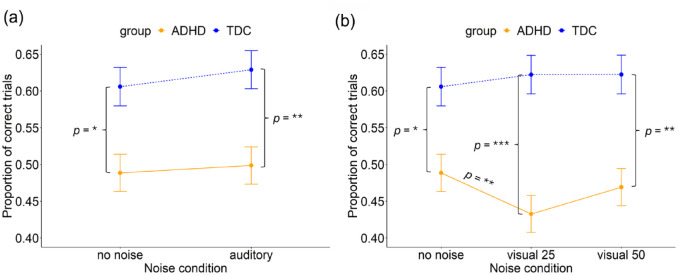
Proportion of correct trials for the ADHD and TDC groups in the (a) no noise and auditory noise condition and, (b). no noise, visual noise at 25% and visual noise at 50% conditions. Error bars represent 95% confidence intervals. *p < .05. **p < .01. ***p < .001.

In the analysis of the proportion of correctly performed MGS trials, the ANOVA found no main effect of noise (*F*(1, 5,570) = 2.68, *p* = .102). However, we observed a significant main effect of group (*F*(1, 95) = 7.06, *p* = .009). No interaction between noise and group (*F*(1, 5,570) = 0.36, *p* = .551) was found, see Figure 10a. The model explained 1.6% of the variance (marginal *R*²) and 22.2% of the variance when accounting for both fixed and random effects (conditional *R*²).

A post hoc analysis indicated a significant difference between the groups in both the no noise condition (β = -.12 (*SE* = 0.05), *z* = −2.4, *p* = .015) and the auditory noise condition (β = -.13 (*SE* = 0.05), *z* = −2.7, *p* = .007). The ADHD group had a significantly smaller proportion of correctly performed trials than the TDC group in both no noise (ADHD: *M* = 0.49, *SD* = 0.50; TDC: *M* = 0.61, *SD* = 0.49) and auditory noise stimulation (ADHD: *M* = 0.50, *SD* = 0.50; TDC: *M* = 0.63, *SD* = 0.48).

##### Visual Noise

A two-way repeated measures ANOVA found no main effect of noise (*F*(2, 8,357) = 2.07, *p* = .127). However, a significant main effect of group (*F*(1, 95) = 10.30, *p* = .002) and a significant interaction between noise and group (*F*(2, 8,357) = 3.40, *p* = .034) was found, see Figure 10b. The model explained 2.4% of the variance (marginal *R*²) and 23.0% of the variance when accounting for both fixed and random effects (conditional *R*²).

A post hoc analysis indicated a significant difference between the groups in the no noise condition (β = -.12 (*SE* = 0.05), *z* = −2.5, *p* = .014), the visual noise condition at level 25% (β = -.18 (*SE* = 0.05), *z* = −3.7, *p* < .001) as well as the visual noise condition at level 50% (β = -.15 (*SE* = 0.05), *z* = −3.1, *p* = .002). The ADHD group had a significantly smaller proportion of correct trials in both no noise (ADHD: *M* = 0.49, *SD* = 0.50; TDC: *M* = 0.61, *SD* = 0.49), visual noise at level 25% (ADHD: *M* = 0.43, *SD* = 0.50; TDC: *M* = 0.62, *SD* = 0.49), and visual noise at level 50% (ADHD: *M* = 0.47, *SD* = 0.50; TDC: *M* = 0.62, *SD* = 0.49).

Additionally, within the ADHD group, significantly lower proportion of correct trials was observed in the visual noise condition at 25% compared to the no noise condition (β = .05 (*SE* = 0.02), *z* = 3.3, *p* = .003), the ADHD group performed worse in the visual noise at level 25% condition.

### Task Assessment

A welch two sample t-test revealed a significant difference between the ADHD and TDC group in how they rated the complete test battery, the rating thus covers both the MGS and PF tasks. The ADHD group found the test battery significantly more boring than the TDC group (ADHD: *M* = 5.54, *SD* = 2.65; TDC: *M* = 3.81, *SD* = 2.37; *t*(95) = −3.40, *p* = .001). A medium to large effect was found (*d* = −0.69, 95% CI [−1.1, −0.27]).

## Discussion

This study explored the impact of auditory white noise and visual white pixel noise stimulation on oculomotor control in children with ADHD and typically developing controls (TDC). The groups performed two oculomotor control tasks that were selected based on their efficacy in distinguishing between ADHD and TDC groups in prior studies (Chamorro et al., 2022; Maron et al., 2021). Participants’ performance on the tasks was measured both with and without noise stimulation.

Building on the Moderate Brain Arousal (MBA) model (Sikström & Söderlund, 2007) and previous research on white noise stimulation (Nigg et al., 2024; Pickens et al., 2019), we hypothesized that (H1) the ADHD group should perform significantly worse than the TDC group in the no noise condition and, (H2) noise stimulation would remove these differences. Specifically, (H3) white noise stimulation would improve performance in the ADHD group and, (H4) decrease performance in the TDC group (see Figure 1 for a schematic representation of our hypotheses).

Taken together, the results did not demonstrate any hypothesized effects of white noise stimulation, whether auditory or visual, on either task; performance did not improve for the ADHD group nor deteriorate for the TDC group. The TDC group outperformed the ADHD group only in two measures in the no noise condition. When there was a significant main effect of noise, it was due to worse performance in the ADHD group. This outcome contrasts recent meta-analyses on the effects of auditory white noise stimulation on working memory performance in individuals with ADHD (Nigg et al., 2024; Pickens et al., 2019). Although inhibitory control is an executive function often impaired in ADHD and closely related to working memory (Pievsky & McGrath, 2018), our results suggest that deficiencies in oculomotor inhibitory control during the prolonged fixation (PF) and memory guided saccade (MGS) task might not be responsive to white noise stimulation.

### H1: TDC Will Outperform ADHD in the no noise condition

In contrast to H1, our results did not support the conclusions drawn by Chamorro et al. (2022) and Maron et al. (2021), who identified the PF task as the most sensitive in differentiating ADHD and TDC regarding oculomotor control. We were unable to find differences in performance between the groups in the PF task without noise. This discrepancy might be attributed to the substantial inter-study variability in the meta-analysis by Chamorro et al. (2022) and the wide confidence intervals reported by Maron et al. (2021). Additionally, in contrast to Chamorro et al. (2022) and Maron et al. (2021), a third review on oculomotor deficiencies in ADHD found no support for any group differences in performance for the MGS and PF tasks (Sherigar et al., 2023). The three reviews differ in the number of publications included, with 31 in Chamorro et al. (2022), 26 in Maron et al. (2021) and 12 in Sherigar et al. (2023), which can explain some of their different conclusions. Thus, there is contradicting evidence of the effectiveness of the PF and MGS tasks to differentiate between the groups. Our results add to this by indicating that the PF task is unsuitable to efficiently identify oculomotor deficiencies in ADHD.

Although our study did not find significant group differences in the PF task, we successfully replicated earlier findings of significant differences in anticipatory saccades in the MGS task in the no noise condition (Chamorro et al., 2022; Maron et al., 2021), supporting H1. We also conclude that the proportion of correct trials is a sensitive measure to differentiate between the groups in the MGS task without noise stimulation.

### H2: Noise Stimulation Will Remove Differences Between ADHD and TDC

Contrary to hypothesis H2, several measures were significantly different between groups in the noise condition(s), both in the PF and the MGS task. For instance, even though there were significant differences between the groups in the no noise condition regarding the number of anticipatory saccades and the proportion of correct trials in the MGS task, these differences were not removed by white noise stimulation. Notably, for certain measures the opposite was found. For example, while no significant group differences in saccadic gain were observed without noise, they emerged in all noise conditions. The fact that neither the auditory nor the visual white noise stimulation removed the observed differences between the groups, and in some cases even introduced a difference, is in contrast to our hypothesis on white noise benefits and previous research within the area (Nigg et al., 2024; Pickens et al., 2019; Söderlund et al., 2021). Reasons for this discrepancy is discussed in more detail below.

### H3 and H4: Noise Stimulation Will Improve ADHD and Impair TDC

In the MGS task there were several instances where the noise did change performance within a group. For instance, the ADHD group exhibited a significant decrease in saccadic gain in the auditory noise condition and the condition with visual noise at 50% compared to the no noise condition. This means that the participants in the ADHD group made shorter saccades toward the target and thereby undershot the target to a greater extent compared to the no-noise condition, contrasting hypothesis H3. Similarly, saccadic latencies were significantly higher in the 50% visual noise condition compared to the no noise condition for the ADHD group. The TDC group also had increased saccadic latencies in the visual noise condition at 50%, compared to the noise condition with visual noise at 25%. The result on saccadic latency contradicts H3, that children with ADHD would benefit from white noise stimulation, while it supports H4, that TDC would experience deteriorated performance. In addition, children with ADHD had a smaller proportion of correct trials in the visual noise condition at level 25%, compared to the no noise condition. Taken together, the results on saccadic gain and latency indicate poorer performance during noise stimulation, especially during the visual noise condition at 50%. The beneficial effects of noise stimulation on working memory do not seem to apply to oculomotor performance. Any clinical implications do not seem applicable at this time.

### Why Where There No Effects of Noise?

There may be several reasons why no effect of noise stimulation was found, spanning from task selection, the executive function studied and the nature of the noise, to sample size and group representativity. First, in their review on auditory noise, Pickens et al. (2019) reported that there is support of white noise benefits on working memory performance. However, they also conclude that not all tasks affected by ADHD seem to be improved by white noise stimulation. Thus, white noise might not be effective in improving performance in the tasks used in this study. Another possibility could lie in the executive function investigated, where studies have shown varying results concerning the effect of noise stimulation on inhibition and impulsive behavior. One previous study on impulsive behavior, where children with ADHD had to choose between a smaller reward sooner or a larger reward later, found no beneficial effects of noise stimulation on their choice (Metin et al., 2016) while inhibitory ability, as measured by a go/no-go task, has shown to be improved in children with ADHD by auditory white noise stimulation (Baijot et al., 2016; Helps et al., 2014). Since it is arguable that the go/no-go task targets similar executive functions as the oculomotor inhibitory tasks in this study, we did expect similar effects of white noise stimulation.

The nature of the noise might be another reason for the varying effects of noise stimulation found. On the one hand, both studies from Metin et al. (2016) and Helps et al. (2014) applied auditory white noise stimulation within the range of 75 to 80 dB, which is similar to the level used in this study (78 dB), and the visual noise is similar to that used in Söderlund et al. (2021). On the other hand, details on how noise is generated and delivered in previous studies are sparse, which makes such studies difficult to replicate. There are also different types of noise (e.g., pink, brown) that, in theory should not but in practice could, induce different effects (Nozaki et al., 1999). In light of this, an important contribution of this study is to make all code to generate and present noise publicly available on GitHub (https://github.com/marcus-nystrom/white-noise-exp).

The representativity of our groups may also be a reason for why we are unable to replicate previous findings. For example, when comparing our results with previous studies on PF (Bucci et al., 2014, 2017, 2018), our TDC group seem to perform more intrusive saccades. This might be since we recruited a naturalistic sample of participants. On the other hand, the groups are expected to be representative for ADHD and TDC since significant differences in ADHD symptoms between the groups were confirmed through SNAP-IV analysis. Another possible confounder is the fact that all children had a training session of the MGS task, which might have improved their performance and thus affected our results.

### Data Loss

Significant group differences in data loss were observed, with the ADHD group experiencing more data loss than the TDC group. There are at least two reasons why more data were lost for children with ADHD. Children with ADHD are more hyperactive and impulsive than typically developed children. Hence, blinks could be more frequent and/or longer in this group (Fried et al., 2014). During blinks, the eyelids are closed, and eye tracking fails. In addition, data could be lost since children with ADHD look beyond the trackable range of the eye tracker, at locations outside of the screen area. Notably, the ADHD group rated the tasks as more boring compared to the TDC group, something that may trigger such behavior.

### Limitations

The statistical power of our study is adequate to detect medium-sized effect sizes, but not to detect small effect sizes. Additionally, data were collected from a single site and contain potential sampling biases. There is also a large heterogeneity in ADHD with potential confounding variables or sub types of ADHD that were not explored, such as gender, comorbidities, and hyperactivity vs. inattention (Reimann et al., 2024). Nevertheless, significant differences in ADHD symptoms between the groups were confirmed through SNAP-IV analysis and groups are expected to be representative for ADHD and TDC. Another possible limitation is that the required medication discontinuation was not physically inspected, using for example a blood test to measure the level of methylphenidate (MPH) in the blood. However, common practice is to use a washout period of 24 hours. See Quinn et al. (2007) for levels of MPH in the blood stream after administration. Participants and their legal guardians were thoroughly informed that non-adherence to medication discontinuation was an exclusion criterion, and all participants were reminded of this the day before their participation in the study.

## Conclusions

This study found no beneficial effects of auditory and visual white noise stimulation on oculomotor control in children with ADHD or TDC, and thus questions whether white noise stimulation improves oculomotor performance for children with ADHD in tasks that target oculomotor inhibitory control.
